# Complete Genome Sequence of the Highly Virulent *Aeromonas hydrophila* AL09-71 Isolated from Diseased Channel Catfish in West Alabama

**DOI:** 10.1128/genomeA.00450-14

**Published:** 2014-05-22

**Authors:** Julia W. Pridgeon, Dunhua Zhang, Lee Zhang

**Affiliations:** a Aquatic Animal Health Research Unit, USDA, ARS, Auburn, Alabama, USA; b Genomics and Sequencing Laboratory, Department of Entomology and Plant Pathology, Auburn University, Auburn, Alabama, USA

## Abstract

*Aeromonas hydrophila* AL09-71 was isolated from diseased channel catfish in west Alabama during a 2009 disease outbreak. The full genome of *A. hydrophila* AL09-71 is 5,023,861 bp. The availability of this genome will allow comparative genomics to identify genes involved in pathogenesis or immunogens for the purpose of vaccine development.

## GENOME ANNOUNCEMENT

Gram-negative bacterium *Aeromonas hydrophila* is the causative agent of motile aeromonad septicemia (MAS) (1) or epizootic ulcerative syndrome (2) in fish. Red sores, necrosis, ulceration, and hemorrhagic septicemia are typical symptoms of MAS (3). MAS disease outbreaks in channel catfish have led to an estimated loss of more than $3 million in 2009 in west Alabama (4–8). The complete genome of one of the virulent epidemic isolates (*A. hydrophila* ML09-119) from west Alabama has been recently published (8). Lateral genetic transfer has been implicated as the molecular basis of the emergence of an *A. hydrophila* epidemic outbreak (9). Another strain *A. hydrophila* AL09-71 was also collected from channel catfish in west Alabama during the 2009 disease outbreak (5, 6). Virulence studies revealed that *A. hydrophila* AL09-71 was highly virulent to channel catfish (5, 6). Through mass spectrometry, 23 proteins were identified in toxic fractions of the extracellular products of *A. hydrophila* AL09-71 (10). However, it was unknown how many virulence related genes existed in the genome of *A. hydrophila* AL09-71. Whole genome sequencing will enable the revelation of the complete list of potential virulence factors in *A. hydrophila* (11). Therefore, the complete genome sequence of *A. hydrophila* AL09-71 was determined in this study.

The genome of *A. hydrophila* AL09-71 was sequenced using an Illumina 1500 Hiseq platform. BioNumerics (Applied Maths) was used to assemble a total of 24,036,568 sequence reads with an average length of 100.24 bp (estimated 479×coverage). Using the west Alabama epidemic isolate *A. hydrophila* ML09-119 genome (accession no. CP005966) as reference, the assembled genome of *A. hydrophila* AL09-71 is 5,023,861 bp with a G+C content of 60.8%. RNAmmer (12) predicted 11, 10, and 10 copies of 5S RNA, 16S RNA, and 23S RNA, respectively, in the genome of *A. hydrophila* AL09-71, similar to that in the genome of *A. hydrophila* ML09-119 (8). A Vista comparative genomics tool (13) revealed 99.5% conserved intervals between the two genomes, with 17 intervals (a total of 4,938,481 bp) sharing 100% identities. The RAST server (14) predicted 4,489 coding sequences belonging to 533 subsystems, including 84 involved in virulence, disease, and defense. In addition, the RAST server identified the following subsystems: 192 in cell wall or capsule, 22 as phages, prophages, transposable elements, or plasmids, 183 in membrane transport, 56 in iron acquisition and metabolism, 127 in motility and chemotaxis, 295 as cofactors, vitamins, prosthetic groups, or pigments, 38 in potassium metabolism, 123 in DNA metabolism, 224 in RNA metabolism, 240 in protein metabolism, 45 in nitrogen metabolism, 37 in sulfur metabolism, 44 in phosphorus metabolism, 127 in nucleosides and nucleotides, 44 in cell division and cell cycle, 100 in regulation and cell signaling, 6 in secondary metabolism, 9 regulons, 136 in fatty acids, lipids, and isoprenoids, 3 in dormancy and sporulation, 171 in respiration, 159 in stress response, 437 in amino acids and derivatives, and 466 in the carbohydrates subsystem.

### Nucleotide sequence accession number. 

The complete genome sequence of *A. hydrophila* AL09-71 was deposited at DDBJ/EMBL/GenBank under the accession no. CP007566.
